# Creating and validating a scholarly knowledge graph using natural language processing and microtask crowdsourcing

**DOI:** 10.1007/s00799-023-00360-7

**Published:** 2023-04-05

**Authors:** Allard Oelen, Markus Stocker, Sören Auer

**Affiliations:** TIB – Leibniz Information Centre for Science and Technology, Hannover, Germany

**Keywords:** Crowdsourcing microtasks, Knowledge graph validation, Scholarly knowledge graphs, User interface evaluation

## Abstract

Due to the growing number of scholarly publications, finding relevant articles becomes increasingly difficult. Scholarly knowledge graphs can be used to organize the scholarly knowledge presented within those publications and represent them in machine-readable formats. Natural language processing (NLP) provides scalable methods to automatically extract knowledge from articles and populate scholarly knowledge graphs. However, NLP extraction is generally not sufficiently accurate and, thus, fails to generate high granularity quality data. In this work, we present TinyGenius, a methodology to validate NLP-extracted scholarly knowledge statements using microtasks performed with crowdsourcing. TinyGenius is employed to populate a paper-centric knowledge graph, using five distinct NLP methods. We extend our previous work of the TinyGenius methodology in various ways. Specifically, we discuss the NLP tasks in more detail and include an explanation of the data model. Moreover, we present a user evaluation where participants validate the generated NLP statements. The results indicate that employing microtasks for statement validation is a promising approach despite the varying participant agreement for different microtasks.

## Introduction

Every year, the number of published scholarly articles grows [1], making it increasingly difficult to find and discover relevant literature. Methods and tools to organize scholarly knowledge are therefore becoming more important. One of the key challenges is the ability of machines to interpret the knowledge published in scholarly articles. Without *machine actionable* scholarly knowledge, machines are severely limited in their utility to effectively organize this knowledge [2]. Knowledge graphs are a possible solution, as they enable knowledge to be represented in a machine-actionable manner. Once scholarly knowledge is machine readable, a variety of new knowledge discovery methods become available. For example, it will then be possible to find articles that employed specific methods yielding specific results or to automatically generate benchmarks of methods ranked by their performance. There are numerous potential use cases for exploiting machine-readable scholarly knowledge that could ultimately lead to new ways of doing science.

**Fig. 1:**
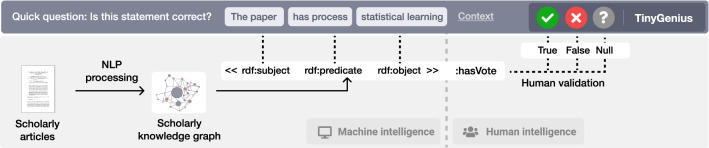
Graphical abstract. Workflow of the TinyGenius methodology. Scholarly articles are processed by NLP tools to form a scholarly knowledge graph (*machine intelligence* part). Afterwards, the extracted statements are validated by humans by means of microtasks (*human intelligence* part). User votes are stored as provenance data as part of the original statements

For centuries, scholarly knowledge has been communicated in a narrative document-based and largely unstructured form [3]. In order to create a scholarly knowledge graph, structured knowledge has to be either extracted from the unstructured documents or produced directly upfront in the research workflow [4]. There are different strategies to support the extraction process. It is possible to manually extract structured knowledge. Although this will likely result in high-quality data, the approach does not scale well. Another approach is to automatically extract structured knowledge using machine learning techniques. Specifically, natural language processing (NLP) is able to interpret natural language and transform unstructured content into a structured, machine-readable representation. However, NLP tools are not sufficiently accurate to generate a high-quality knowledge graph, in particular, due to the complexity of the conveyed information, the required context-awareness or the varying levels of semantic granularity. Naturally, quality is a crucial aspect for a scholarly knowledge graph to become a valuable resource for researchers. Thus, in this work, we propose a hybrid method where we combine human and machine intelligence via microtasks to create a structured scholarly knowledge graph. This results in a synergy and combines the advantages of each approach, i.e., the quality aspect from *human* intelligence and the scalability aspect from *machine* intelligence.

We present *TinyGenius*, a methodology to create a scholarly knowledge graph leveraging intertwined human *and* machine intelligence. Firstly, NLP tools are used to autonomously process scholarly articles. Secondly, the NLP results are transformed into a paper-centric scholarly knowledge graph. Finally, the statements are presented to humans in the form of microtasks. Humans can vote to determine the correctness of the statements. Votes are stored as provenance data on statement level. Based on the votes, an aggregated score is computed to indicate the correctness of a statement. TinyGenius is specifically designed to be integrated in the Open Research Knowledge Graph (ORKG) [5]. The ORKG aims to describe scholarly knowledge in a structured manner using crowdsourcing, resulting in a scholarly knowledge graph. The task of transforming unstructured into structured knowledge, even with NLP assistance, is a complex and time-consuming endeavour. We, therefore, propose a method that decomposes this large task into a set of microtasks. Once integrated into the platform, microtasks are displayed throughout the web interface. This enables regular visitors to be *content producers* not just *content consumers*. For each NLP tool, a specific microtask is designed. This is to ensure task simplicity and to provide a task that can be answered without contextual knowledge (i.e., without reading the article). A screenshot of the interface showing an example of a microtask is depicted in Fig. 1.

**Fig. 2:**
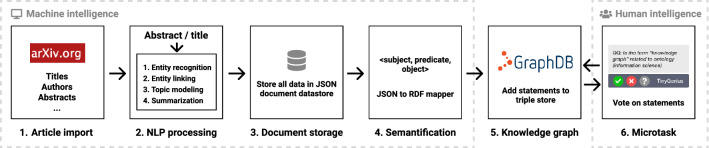
TinyGenius methodology intertwining human and machine intelligence to create a scholarly knowledge graph. ArXiv articles are imported, processed by a set of NLP tools, and the results are stored. From the results, a knowledge graph is generated. Afterwards, humans validate the knowledge graph by means of microtasks

We make the following contributions:

1. The TinyGenius methodology to validate scholarly NLP results using crowdsourced microtasks;
2. A modular architecture to create a scholarly knowledge graph at scale with NLP tools;
3. An evaluation of the methodology and architecture by means of a user study and case study, respectively.

This work is an extension of previous work presented in [6]. In contrast to the previous work, we provide a more comprehensive description of the employed NLP tools and respective tasks. Furthermore, we discuss the user interface in more detail and include a more detailed description of the TinyGenius data model. Finally, we add a user evaluation that includes the discussion of the corresponding results.

## Related work

Large complex tasks can be decomposed into a set of smaller, independent microtasks [7]. These microtasks are context-free, are more manageable, and lead to higher quality results [8]. The context-free setting relates to the absence of required prior task knowledge from a user perspective. While microtasks can be beneficial on an individual level, such as microwork [9], they are commonly performed in a crowdsourced setting by unskilled users [10]. In a crowdsourced setting, a large task, too big in scope for a single person, can be completed collaboratively. Microtask crowdsourcing has been successfully employed for various tasks, for example, writing software programs [7], validating user interfaces [11], labeling machine learning datasets [12], ontology alignment [10], and knowledge graph population [13].

Machine learning tools can process data at scale without the need for human assistance. Therefore, such tools are especially suitable to handle large quantities of data, such as scholarly article corpora. The natural language processing (NLP) domain focuses specifically on understanding natural language for machines [14]. In our methodology, we employ a set of five NLP tools to process scholarly article text. These tools perform four different NLP tasks, which we will now discuss in more detail.

- First, *Named Entity Recognition* (NER) is a task to identify entities within text belonging to a predefined class [15]. For example, the task of identifying the classes “materials” and “methods” within a scholarly article.
- Second, *Entity Linking* is the task of linking entities to their respective entry in a knowledge base [16]. This includes the task of entity disambiguation, to ensure entities are not only syntactically but also semantically the same. For example, the entity “Python” can be linked both to the animal and the programming language. The context determines which link is correct.
- Third, *Topic Modelling* is the task to identify and distinguish between common topics occurring in natural text [17]. This allows for classifying papers based on their mutual topics.
- Finally, *Text Summarization* is the task of compressing text into a shorter form, while preserving the key points from the original text [18].

Knowledge Graphs comprise triple statements according to the W3C Resource Description Framework (RDF) [19]. By using a standardized data representation model, the data interchange between machines is facilitated. This increases the machine actionability of the data, which is defined as the ability for machines to interpret the data without the need for human intervention [20]. RDF data can be queried using the SPARQL language [21]. A key aspect of our approach is storing the user votes as provenance data to statements in the knowledge graph. This means not only a final correctness score of a statement is available, but also the underlying information used to determine the score. Among others, the provenance data include the votes, contextual information, and confidence score of the NLP tool. There are different approaches to store provenance data in RDF [22], for example, standard reification, singleton properties [23], named graphs [24], and via RDF* [25]. We adopted the RDF* representation as this provides a method that scales well and, compared to the other approaches, provides improved comprehensibility for SPARQL queries.

## Architecture and NLP

We now discuss the TinyGenius methodology. First, we describe the technical infrastructure responsible for data storage and processing. Second, we explain the user interface in more detail.

### Technical infrastructure

One of the key benefits of using NLP tools to process data is the ability to perform this analysis at scale. Therefore, the infrastructure is designed to handle large quantities of data while still having a well performing system. Among other things this includes query response time and system resource utilization. We outline the methodology depicted in Fig. 2:

1. In the first step, the complete metadata corpus from the open-access repository service arXiv^1^ is imported. This includes article titles and abstracts. To reduce the required computational resources and ensure a consistent level of semantic granularity, only paper titles and abstracts are processed by NLP tools (i.e., the full-text is excluded).
2. Afterwards, the papers are processed by different NLP tools, which we discuss in Sect. 3.2.
3. In the third step, the output of the paper imports process, and the resulting data from the NLP tools are stored in a document-based JSON data store. Notably, the NLP results are stored in their native data model and are not transformed to make them suitable for knowledge graph ingestion.
4. The semantic transformation process takes place in the fourth step, i.e., semantification. This step converts the native NLP data models to a triple format, as required by the RDF data model. The original data from step three remains available in the original JSON data store. This allows to create a different mapping from the NLP models to RDF at any time in the future, and it separates the concerns between data processing and data modelling.
5. In the fifth step, the data are ingested in a triple store. As discussed previously, we adopted an RDF* provenance data model. Therefore, a GraphDB^2^ triple store is used, which supports RDF* natively. The data model, including an example of data provenance statements, is depicted in Fig. 4. To increase machine actionability, existing ontology concepts are used when possible.

### NLP tools

We employed a set of five different NLP tools to process the articles. The TinyGenius methodology itself is not limited to this set of tools and can be easily extended with other NLP tools. The tools are listed in Table 1. The selected tools provide a representative sample of different NLP tasks. The CSO classifier takes an article abstract as input and outputs extracted topics. This classifier is a domain-specific model designed to classify Computer Science abstracts [26]. The related microtask asks users whether the extracted topic is indeed relevant for the paper. The Ambiverse Natural Language Understanding (NLU)^3^ [27] tool links entities found in a text to a corresponding entry in Wikidata [28]. The microtask is related to determining whether the concept is correctly linked. Users can visit the corresponding Wikidata page to determine the correctness. The Abstract Annotator extracts four classes from paper abstracts: data, material, method, and process [29]. The related microtask lets users validate whether the entity indeed belongs to the selected class. The Title parser is similar to the Abstract annotator, but focuses specifically on titles, which typically follow certain conventions exploited by this tool. Finally, the Summarizer takes an abstract as input and summarizes that into a text piece of maximum 120 characters. The microtask asks users to indicate whether the generated abstract is indeed a reasonable summary.

**Fig. 3:**
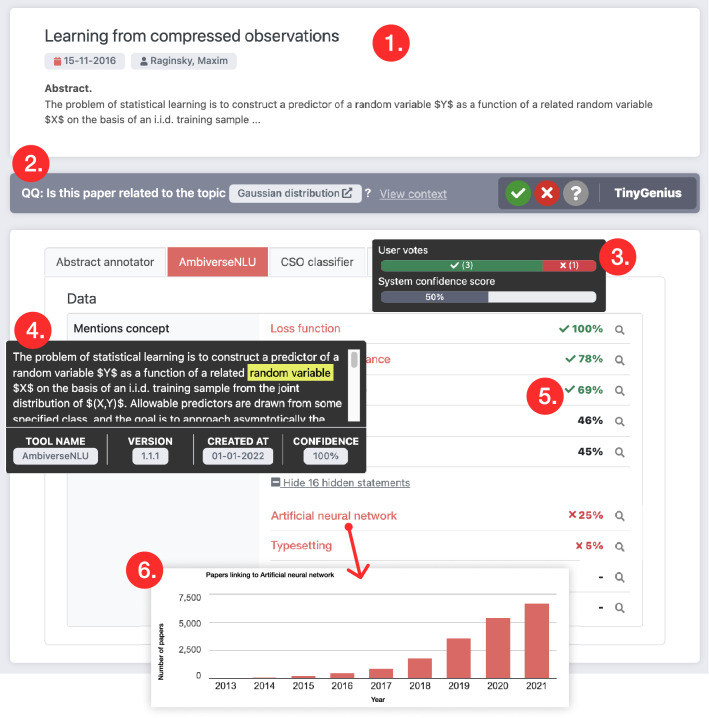
View paper page, showing the integrated voting widget and NLP statements. Node 1 displays the metadata related to the selected paper. Node 2 shows the voting widget. Node 3 is the score tooltip. Node 4 shows a tooltip that displays the context and provenance data related to a single statement. Node 5 lists the NLP-generated statements grouped by the tool. Finally, node 6 shows the use of a resource grouped by year, which is displayed when clicking on a resource

**Table 1:** List of employed NLP tools and their corresponding task and scope. The question template shows how the microtask is presented to the user

Tool name	NLP task	Scope	Question template
CSO classifier	Topic Modelling	Domain-specific	Is this paper related to the topic {topic}?
Ambiverse NLU	Entity Linking	Generic	Is the term {entity} related to {wikidata concept}?
Abstract annotator	Named Entity Recognition	Domain-specific	Is this statement correct? This paper {type} {entity}
Title parser	Named Entity Recognition	Domain-specific	Is {entity} a {type} presented in this paper?
Summarizer	Text Summarization	Generic	Does this summarize the paper correctly?

## Microtask crowdsourcing user interface

The user interface consists of two main components: the voting widget and the view paper page. The voting widget is self-contained, meaning that it contains all the required context to perform the microtask. The view paper page integrates the voting widget for the displayed paper.

### Voting widget

The voting widget is the key interface component and integrates the microtasks to perform the NLP validation. It is displayed in Fig. 3. The widget is self-contained, modular, and is designed to be integrated into a scholarly knowledge web platform. Each NLP tool has a different question template, as listed in Table 1. This question template is used to display the microtask in the widget. The widget itself displays the context required to make an informed decision about the correctness of the statement. In most cases, the context displays an excerpt of the abstract and highlights the words used by the NLP tool to extract the data. Finally, users are able to vote about the correctness. A vote can either be correct, incorrect, or unknown. After a user has voted, a positive affirmation (e.g., “Good job!” or “You rock!”) is displayed to encourage the user to continue with the next statement. The next statement is automatically displayed after voting. Statements are selected in random order, and statements are only displayed once to a specific user.

### View paper page

Fig. 3 shows a screenshot of the view paper page. It shows how a single paper is displayed when integrated within the Open Research Knowledge Graph (ORKG) [5]. All data displayed on the page are coming from the TinyGenius knowledge graph and are fetched using SPARQL. The previously discussed voting widget is also displayed on this page. A score is displayed for each listed statement, indicating how reliable a statement is. When hovering over the score a tooltip becomes visible, explaining how the score is determined. This is an aggregation of user votes, counting for 75% of the score, and the system’s confidence level, counting for the remaining 25% for the score. By default, statements with a score below a certain threshold (40%) are hidden. Finally, the context tooltip shows statement provenance data. This includes an excerpt from the abstract used by the NLP tool to generate the result. Furthermore, additional data related to the tool, version, and date are displayed. The listed statement resources link to a page that shows the use of the respective resource over the years (see node 6 in Fig. 3).

## Evaluation

The objective of the evaluation is twofold. Firstly, we conduct a data evaluation to gather general statistics about our approach and to assess the technical performance. Secondly, we use a sample of the data generated in the first evaluation to conduct a user study. The user study is an exploratory evaluation, and its results are used to guide further development and to assess the feasibility of the approach.

**Table 2:** Overview of the data evaluation statistics

Description	Measure
*General statistics*	*Number*
Processed articles	95,376
Triples metadata	1,521,492
Triples provenance	47,595,706
Triples total	65,608,902
Average number of triples per article	688
*Processing time*	*Seconds*
CSO classifier	27,803
Ambiverse NLU	137,060
Abstract annotator	62,056
Title parser	87
Summarizer	N/A

### Data evaluation

We imported the arXiv corpus and processed a subset with selected NLP tools. All articles classified as “Machine Learning” by arXiv^4^ are processed. This results in a total amount of 95, 376 processed articles, which is approximately 5% of the complete arXiv corpus. We consider this a sizable amount to estimate statistics such as processing time per article, number of extracted statements per article, and to determine the performance of the setup. We chose the machine learning field because several NLP tools are trained specifically on machine learning abstracts. The processing time in seconds per NLP tool is listed in Table 2. In addition to the total number of triples, an approximation of the number of metadata and provenance triples is listed. The tools ran on a machine with 40 CPU cores and no dedicated GPUs. As the summarizer tool requires GPUs to run efficiently, we did not apply this tool to the entire dataset. Instead, we ran the summarization tool for the sample of articles used in the user evaluation.

### Performance evaluation

**Fig. 4:**
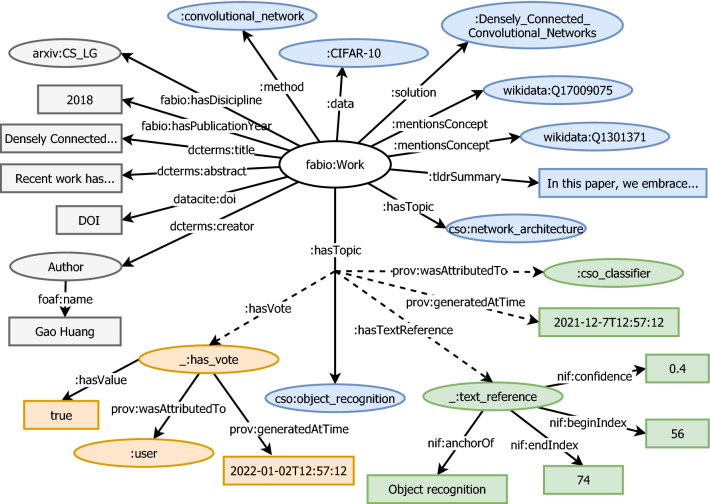
Example of paper subgraph including provenance data. Grey nodes represent metadata related to the work. Blue nodes indicate NLP-generated knowledge for the respective paper. The dashed lines represent provenance data for the statement. In this example, green nodes indicate provenance data related to the context (i.e., the explanation of how to NLP tool came to this result). The orange nodes represent the data for a single user vote on the statement

To determine the performance of the triple store with the ingested data, we now present three prototypical SPARQL queries and their respective execution time. The results of running the queries are listed in Table 3. The queries also demonstrate how data can be accessed via our data model, as outlined in Fig. 4. The previously presented user interface uses the listed queries to render the paper data, and therefore, the queries are representative for use in an actual system.

The queries are executed on the same machine as used for the NLP processing. Furthermore, the same data are used to query data as listed in Table 2 (i.e., 65,608,902 triples). Query 1 demonstrates how statements can be retrieved based on the NLP tools used to generate them. Query 2 queries all available data for a single paper. A similar query is used in the user interface to display paper data. Finally, Query 3 counts all articles that are related to a specific resource, grouped by year. The plotted result of this query is displayed in Fig. 3 node 6. The resulting execution time indicates that the triple store performs well, even for more complex queries such as aggregating data from all papers in the graph.

**Table 3:** Results of the performance evaluation for running three different queries

Query	Execution time (s)	Retrieved triples
Query 1	0.1	23
Query 2	0.2	653
Query 3	0.4	15


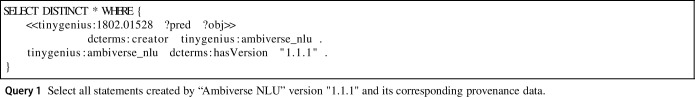



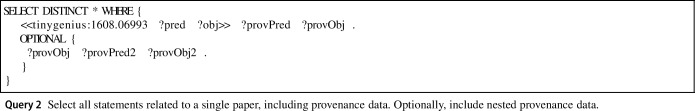
 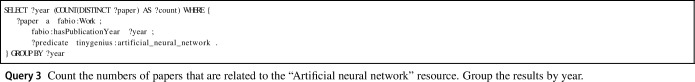


### User evaluation

We created an online evaluation environment to evaluate the TinyGenius approach. We focused on evaluating the voting widget, specifically targeting the microtasking aspect of our approach.

#### Experimental setup

In total, we recruited 11 participants. All participants are researchers with a Computer Science background. We adopted convenience sampling for the recruiting process. Participants were asked to visit the online evaluation environment which guided them through the evaluation. An interactive help guide explained the objectives of the evaluation and what was expected from the participants. Additionally, several interface components were highlighted and explained in more detail. The appearance of the evaluation interface mimics the design of the scholarly platform where we plan to integrate TinyGenius. Interface components not needed for the evaluation were either disabled or hidden. This is to ensure participants are not leaving the page and potentially not finishing the study.

Participants were asked to validate 114 statements coming from ten different articles. These articles were sampled from the dataset generated in the data evaluation section. The ten most popular articles from this set are selected for evaluation.^5^ The rationale for selecting popular articles is that those articles are likely to contain relevant knowledge, hence the popularity of the articles. From the selected articles, statements were randomly selected and limited to three statements per NLP tool. The random statement selection simulates a realistic scenario where NLP tools generate statements that are possibly clearly wrong, clearly right, or ambiguous and therefore hard to assess for correctness. Notably, the participants were not selected based on their knowledge of machine learning. Our assumption is that this knowledge is not required to perform the majority of the microtasks as most tasks consist of relatively simple questions that do not require deep domain knowledge.

After participants completed the microtasks, they were asked to fill out a questionnaire. This questionnaire consisted of 35 questions of which most are answered with a 5-point Likert scale, ranging from “strongly disagree” to “strongly agree”. The questionnaire has several objectives, including determining the attitudes towards the overall voting approach, assessing how participants feel about the specific microtasks, and gathering additional feedback. Furthermore, the questionnaire contained two standardized evaluation methods. System Usability Scale (SUS) [30] questions are included to determine the usability of the voting widget interface. Additionally, questions from the NASA Task Load Index (TLX) [31] are included to assess the perceived task load by the participants.

**Fig. 5:**
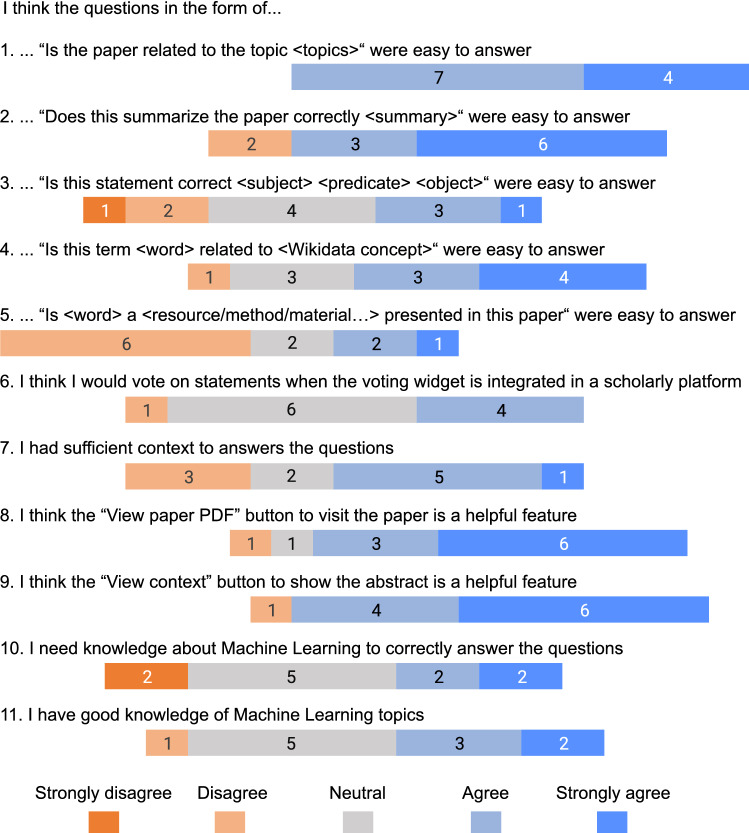
Questionnaire results from the questions with a Likert scale. The first five questions relate to the specific NLP tools. The remaining questions are either about the use of the voting widget or the participants’ knowledge of machine learning

**Fig. 6:**
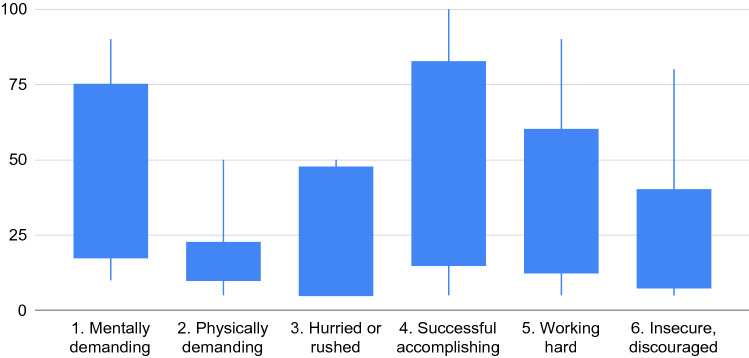
Outcomes of the NASA Task Load Index (TLX), using non-weighted questions. Higher values indicate more task load. Line endings represent minimum and maximum values. Boxes represent the first and third quartile

#### Evaluation results

A set of answers from the questionnaire is plotted in Fig. 5. The first five questions are related to the five NLP tools. As can be observed from the results, microtasks related to the CSO classifier (question 1), Summarizer (question 2), and Ambiverse NLU (question 4) are considered relatively straightforward by the participants. On the other hand, microtasks related to the Abstract annotator (question 3) and Title parser (question 5) are considered more difficult. Most likely this type of task requires more domain knowledge, and possibly more knowledge about the actual article, as participants have to decide whether a certain term is correctly classified. Furthermore, the results suggest that most participants had sufficient context to answer questions and that the “View paper PDF” button, and especially the “View context” feature, were appreciated (question 8 and 9, respectively). Question 10 and 11 give an indication of the required domain knowledge according to the participants. While participants are mostly neutral about the question whether domain knowledge (in this case Machine Learning knowledge) is required, some participants considered themselves knowledgeable regarding this domain. This can introduce bias regarding the task complexity, as knowledgeable participants are more likely to underestimate the required skills.

**Fig. 7:**
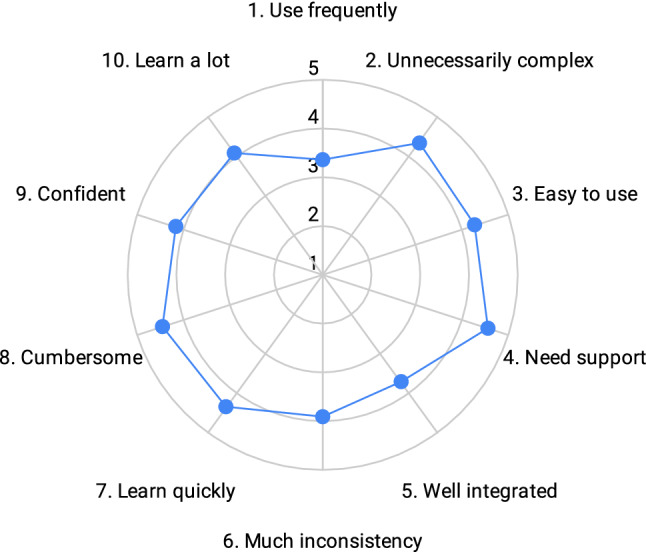
Outcomes of the System Usability Scale (SUS) questions. Questions are abbreviated, and the full list of questions is provided by Brooke et al. [30]. Answers are normalized so that higher scores mean better usability (SUS uses alternating positive and negative questions to reduce response bias)

The TLX outcomes are shown in Fig. 6. The average task load is 33.79 (SD = 17.43), which is low compared to the average of 45.29 determined by Grier [32] (lower is better). The standard deviation is relatively high, indicating that some participants considered the task more demanding than others. This also becomes apparent from the question related to the time needed to finish the evaluation. While some indicated to be finished within 10 to 20 min, others needed considerably more time, between 30 and 60 min, and one participant more than 60 min. The perceived machine learning knowledge of participants is displayed in Fig. 5 question 11. However, the time required to finish the task does not seem to correlate with their knowledge of machine learning. The results from the SUS evaluation are displayed in Fig. 7. The average SUS score is 78.18 (SD = 11.68) which is considered “good”.

Finally, we evaluated the voting data produced by the participants. In total, 1, 254 votes were collected. From these, 122 votes were “unknown”, meaning that participants were not sufficiently confident to vote. To assess the agreement among participants, we determined the inter-rater reliability from the voting data. The results are listed in Table 4. Specifically, we calculated Krippendorff’s alpha [33], which is used as a reliability coefficient when there are multiple observers (i.e., participants) and missing data. In our case, the “unknown” vote is considered missing data. The statistic ranges from $-1$ to 1, where 1 means unanimous agreement, 0 no agreement apart from chance, and $-1$ means inverse agreement [34]. We calculated the agreement per NLP tool. Interestingly, a relatively large difference between the tools can be observed. More agreement is found for the CSO classifier and Ambiverse NLU, and less agreement for the Abstract annotator and Title parser. This is in line with the results from the participants’ own judgments related to the difficulty per NLP tool (questions 1 to 5 in Fig. 5). The summarization tool has a negative agreement, indicating that this type of task in its current form is not producing meaningful results.

## Discussion

We now discuss our methodology and evaluation in more detail. Furthermore, we discuss the limitations and future work.

### System usage

The evaluation results indicate that the presented method is promising and the proposed setup and infrastructure are suitable for the task. When the methodology is deployed in a real-life setting, the knowledge graph quality can be substantially improved. Over time, more visitors will vote on the presented statements, increasing the overall data accuracy. The user votes are stored as provenance data on the statement level, providing the opportunity for downstream applications to decide how to incorporate the validation data. Incorrect data can simply be filtered out, but it is also possible to perform more complex analysis on the validation data.

The generated knowledge graph comprises both correct and incorrect statements, no matter if they are considered to be invalid according to user votes. The adds an overhead while querying the data, as the respective provenance data has to be queried as well, in order to determine the correctness of a statement. However, we deem the RDF* syntax to be sufficiently intuitive to query provenance data, which reduces the overhead from a user perspective. Another possibility is to include an aggregated correctness score for each statement which makes it possible to filter out incorrect statements, without having to query the provenance data of a statement. We did not include such a mechanism in the current setup, as the usefulness of such solutions heavily relies on the use case of the data. As mentioned before, in the end downstream applications can decide how to handle incorrect statements.

**Table 4:** Aggregated results from the voting task, grouped by NLP tools. Krippendorff’s alpha indicates the agreement among the participants

Tool name	Krippendorff’s $\alpha$	Votes
CSO classifier	0.31	330
Ambiverse NLU	0.36	330
Abstract annotator	0.021	330
Title parser	0.14	154
Summarizer	$-$0.032	110

The data and performance evaluations show that the current setup performs well and is able to handle the scale of the knowledge graph without major issues. Naturally, more complex queries will result in increased execution time, especially when the knowledge graph grows in size. However, we limited our performance evaluation by running queries that are needed to render the user interface. One of the requirements for web applications is that loading times should be low, preferable below two seconds, which is considered a tolerable waiting time for web users [35]. As the evaluation results indicate, it is indeed possible to load the page within this time frame. Here, we specifically focused on machine learning articles from the arXiv corpus. Some of the selected NLP tools are domain models, specifically trained on Computer Science. However, our approach is not limited to this domain. By design, the system is modular and can be generalized to support other domains and NLP tools.

**Fig. 8:**
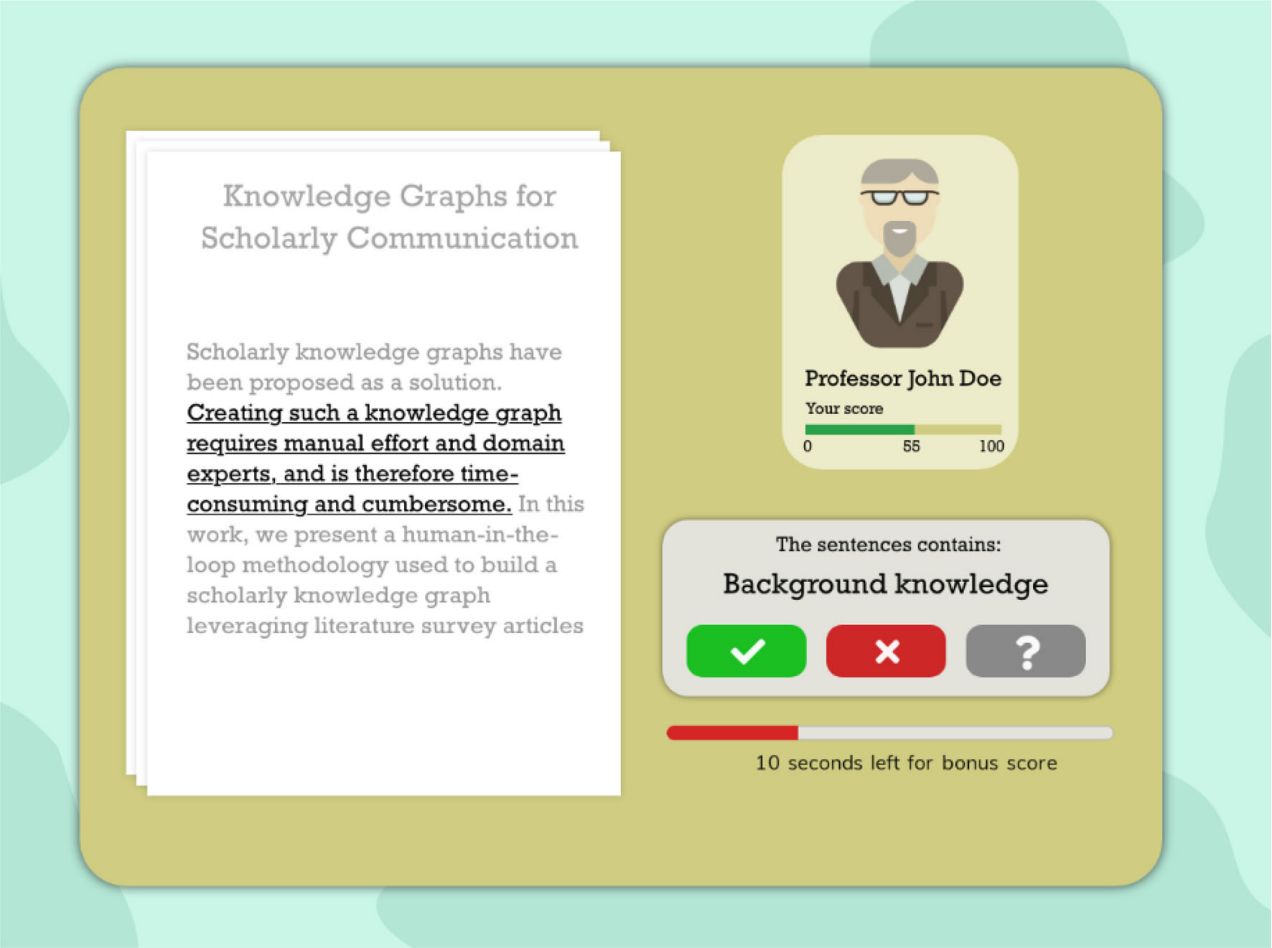
Prototype of a gamification user interface using the TinyGenius methodology. The player has to advance in their scientific career by solving the displayed tasks

The generated knowledge graph provides opportunities for multiple data consumption use cases. For example, by linking concepts between articles, scientometrics can be conducted on the data. This includes methods to plot research trends over time or to find related papers by means of commonly used materials and methods. By making the data accessible via SPARQL, we provide a powerful interface to support such use cases. Query 3 is an example for research trend analysis. Other use cases include data exploration interfaces, such as a dynamic faceted search to more effectively find research articles. Due to the availability of structured data, it becomes possible to perform precise search queries. Implementing data consumption use cases is out-of-scope for this work and will be part of future work.

### System usability

The user evaluation indicated that the usability of the voting widget is good. This finding is also supported by the additional results from the questionnaire. The preliminary user evaluation gives an impression of the overall approach and guides further development. The inter-rater reliability outcomes are relatively low. This is expected as annotators were not trained and had only little information on how to perform the task. If more extensive annotation guidelines were provided, the agreement among annotators is expected to increase. However, this goes against the principle of having low-context and easy-to-perform microtasks. Furthermore, the agreement seems to also depend on the type of microtask. Entity linking and topic modelling tasks are arguably more straightforward than named entity recognition tasks, which are generally more ambiguous and therefore harder to evaluate. Additionally, the summarization task seems unsuitable for our microtask. Often, a summary is not considered completely wrong or right, which makes it unsuitable for a binary voting task. For future work, we plan to extend the voting widget with a score slider to allow for interval scoring for this specific task.

### Limitations

Due to the low number of participants, no statistical conclusions can be drawn from the results. Consequently, we cannot make definite conclusions about how suitable the selected microtasks are to generate a high-quality knowledge graph. However, we selected our evaluation methods based on the low number of participants, for example, the System Usability Scale (SUS) is a reliable statistic for small sample sizes [36]. The homogeneity of the population (i.e., all participants had a Computer Science background) makes further evaluation necessary. Yet, we deem this a realistic setup, since the microtasks can be allocated to crowd-workers with a respective background. Therefore, although the microtasks generally do not require domain knowledge, high-level knowledge can be expected from the crowdsourcing participants due to predefined task allocation. Finally, certain domains are presumably more suitable for crowdsourcing microtasks than others. In this work, we did not consider different fields, which we consider a limitation of this work.

### Future work

The current setup implements the voting widget within a scholarly knowledge graph infrastructure. However, for future work, we envision the widget to be implemented within external systems as well. Due to the simplicity of the task, the widget can be integrated into third-party systems and different settings. For example, arXiv provides a section “arXivLabs” where additional information related to a publication can be displayed. This section is suitable for TinyGenius-related data as well, providing opportunities to collect additional user votes. Furthermore, data can be collected in a casual microtasking setting [37]. For example, data can be collected via Twitter where questions are asked in Tweets and answers can be provided via comments. Although the voting setup will be different than presented within our work, the same underlying knowledge graph and data model can be used.

Additionally, we plan to leverage the TinyGenius methodology within a gamification approach. A prototype of such an implementation is depicted in Fig. 8. Also with this approach, the underlying data model is the same. Completed tasks within the game environment generate voting data for the displayed statements, contributing to the validation of the knowledge within the graph. Compared to the voting widget (displayed in Fig. 3), the gamification interface provides extra incentives for users to vote on statements. By making the correct votes, they make progress in the game and advance to the next levels. Gamification has been applied in businesses, politics, health, and education [38]. We therefore consider a gamified graph validation approach as a promising future research direction.

## Conclusion

We presented TinyGenius, a methodology to validate NLP statements using microtasks. We applied and evaluated TinyGenius in the scholarly domain. The method combines machine and human intelligence resulting in a synergy that utilizes the strengths of both approaches. Firstly, a set of NLP tools is applied to a corpus of paper abstracts. Secondly, the resulting data are ingested in a scholarly knowledge graph. Finally, the data are presented to users in the form of microtasks. By utilizing microtasks, the data are validated using human intelligence. The performance evaluation indicated that the used triple store is able to handle the data quantity without issues. The user evaluation showed that the usability of the system is good. We deem this work to be one of the first, which truly combines human and machine intelligence for scholarly knowledge graph creation and curation. This combination needs much more attention, since there are many important use cases, where machine intelligence alone can (e.g., due to the missing training data) not produce useful results.

## Footnotes

1 https://arxiv.org/.

2 https://graphdb.ontotext.com/.

3 https://github.com/ambiverse-nlu/ambiverse-nlu.

4 arXiv category: Machine Learning (cs.LG).

5 Top 10 based on popularity determined with https://github.com/karpathy/arxiv-sanity-preserver.
